# Level of Unawareness and Management of Diabetes, Hypertension, and Dyslipidemia among Adults in Luxembourg: Findings from ORISCAV-LUX Study

**DOI:** 10.1371/journal.pone.0057920

**Published:** 2013-03-04

**Authors:** Ala’a Alkerwi, Sybil Pagny, Marie-Lise Lair, Charles Delagardelle, Jean Beissel

**Affiliations:** 1 Department of Public Health, Centre de Recherche Public-Santé (CRP-Santé), Strassen, Grand-Duchy of Luxembourg; 2 Service de Cardiologie, Centre Hospitalier du Luxembourg, Luxembourg, Grand-Duchy of Luxembourg; Sapienza University of Rome, Italy

## Abstract

**Background:**

In the absence of evidence-based information, assessment of population awareness and management of diabetes, hypertension and dyslipidemia (treatable and preventable cardiovascular risk factors) are important to halt coronary and cerebrovascular diseases and to improve public health.

**Methods:**

The analysis was based on a nationally representative sample of 1432 adult subjects, recruited for the ORISCAV-LUX survey (2007–2008). Descriptive and multivariable logistic regression analyses were performed. The 10-year Framingham risk score was calculated for each participant who classified at low, intermediate and high risk.

**Results:**

Among the diagnosed cases, 32%, 60%, and 85% were respectively unaware of their diabetes, hypertension and dyslipidemia. Increasing age and BMI were the strongest protective factors against unawareness of hypertension and dyslipidemia. Having a family history decreased the risk of unawareness of hypertension (OR = 0.57; 95% CI 0.36, 0.92; *P* = 0.021), whereas, not having a family doctor increased double-fold the odd of being unaware of hypertension (*P* = 0.048). Poor health perception reduced significantly the risk of unawareness of dyslipidemia (OR = 0.27; 95% CI 0.11, 0.68). Concerning the management, diabetes was markedly better treated than hypertension and dyslipidemia. Among diabetic subjects (constituting 4% of the population), 3% were treated vs. 1% not treated. In contrast, 22% of the hypertensive participants (35% of the population) were not treated vs. 13% treated. Concerning dyslipidemia, only 9% of those with lipid disorder (70% of the population) were under medication vs. 61% not treated. For the treated cases of these pathologies, almost only one-third was under control. Framingham risk of developing CHD within 10 years was moderate to high among 62%, 27%, and 17% of the unaware/untreated diabetic, hypertensive, and dyslipidemic participants, respectively.

**Conclusion:**

The considerable lack of awareness and insufficient management underscore the urgent need for intensive efforts to reduce the gap in prevention strategies, and control of cases according to explicit clinical guidelines.

## Introduction

In Luxembourg, cardiovascular mortality is the first leading cause of mortality, accounting for about one-third of total deaths, with a stable trend during the last decade [1]. Diabetes, hypertension, and lipid disorders commonly co-exist and constitute the most common risk factors for coronary heart disease (CHD). Subjects who suffer from these pathologies are often unaware that they are afflicted until they experience debilitating complications. Patients with silent hypertension associated with dyslipidemia and uncontrolled diabetes are often susceptible for premature myocardial infarction and hemorrhagic stroke. The asymptomatic character of these treatable and preventable risk factors contributes to increasing the incidence of cerebro- and cardio-vascular diseases and of sudden death.

Successful management (treatment and control) of these pathologies depends primarily on sufficient patient awareness of their existence to achieve best treatment compliance. Good clinical control and self-care (regular consultations, self-measurement of glycemic level and blood pressure) can delay complications and maintain quality of life.

A recent patient-based study in Luxembourg has demonstrated a poor awareness of cardiovascular risk factors in high-risk patients who underwent coronary angiography, with significant social inequalities [2]. Moreover, the first nationwide population-based ORISCAV-LUX survey (2007–2008) revealed a high prevalence of these cardiovascular risk factors among a random representative sample of presumably healthy adults; dyslipidemia (69.9%) was the most predominant cardiovascular risk factor, followed by hypertension (34.5%), and diabetes (4.4%) [3].

From a clinical and public health standpoint, knowledge of the current population awareness of these cardiovascular risk factors is important, not only to improve their management, but also to allocate appropriate health care resources, and to address targeted health education messages. In this study, our objectives were to 1) assess the population level of unawareness of each condition, namely, diabetes, hypertension, and dyslipidemia, 2) identify the potential determinants of lack of awareness, 3) evaluate the level of management for the three pathologies and 4) provide information about the 10-year risk prediction of CHD among the unaware (untreated) groups, using a recent nationwide representative sample. This report aims to address the challenges faced by our public health decision-makers and healthcare professionals working in primary care settings.

## Methods

### Study Population

The ORISCAV-LUX is a cross-sectional population-based cardiovascular risk factors survey, conducted between November 2007 and January 2009 in Grand-Duchy of Luxembourg. A representative random sample of 4 496 non-institutionalized subjects residing in Luxembourg, stratified according to gender, age (5-year categories) and geographic district (Luxembourg, Diekirch and Grevenmacher) was drawn from the regularly updated national health insurance registry, with a 98% social coverage rate. The minimal necessary representative sample size was calculated to 1 285 subjects to ensure statistical power, i.e. to ensure a statistical precision of at least 2% for the estimation of the prevalence of the risk factors at the 95% confidence level. However, a total of 1 432 subjects took part in the survey, yielding a participation rate of 32.2%. The distribution of selected subjects in each stratum was proportional to their distribution in the source population. A comprehensive description of the protocol, survey design, sampling method and sample representativeness has been published in previous reports [3], [4]. Briefly, selected persons were invited through an official letter followed by a phone contact to confirm the appointment. The trained research nurses either visited participants in their households or invited them to the nearest study investigation center. At the time of interview, the participants initially signed the informed consent form and then filled in the auto-administered questionnaire, to collect data on subject’s demographic, socioeconomic characteristics, history of hypertension, diabetes and dyslipidemia and the use of respective medications.

Minimum 8-h fasting blood samples were analyzed for glucose, total cholesterol (T-C), HDL cholesterol (HDL-C) and triglycerides (TG). For blood pressure measurement, systolic blood pressure (SBP, mmHg) and diastolic blood pressure (DBP, mmHg) were measured at least 3 times with a minimum of 5-min interval, by using Omrom® MX3 plus automated oscillometric Blood Pressure Monitor (O-HEM-742-E) (Matsusaka, Japan) [5], according to standard operating procedure. Measurements were only performed after the participants had been seated for at least 5 minutes after questionnaire completion and at least 30 minutes after blood intake and refrained from smoking. The average of the last 2 readings was used in the analysis. The rationale behind discarding the first reading was based on literature review; to avoid false positive classification for reactive subjects with otherwise normal blood pressure fluctuations, and because taking the average of the second and third readings in case of triplicate measurements may best predict the awake SBP [6].

### Definitions

#### Diabetes

Presence of diabetes was based on self-reporting of anti-diabetic medications, and/or fasting plasma glucose value (FPG)≥126 mg/dl [7]. For glycaemic control, persons with glycosylated hemoglobin (HbA1c) level <6.5% were considered as controlled [8], [9]. If the participants reported that they had ever been told by their doctor that they had diabetes and/or if they self-reported anti-diabetic medication intake, they were considered as clinically diagnosed (aware) of their diabetes. All clinically undiagnosed or non-treated participants were thus considered as unaware of their diabetes.

#### Hypertension

Hypertension was defined as a mean SBP≥140 mmHg and/or DBP≥90 mmHg, and/or the use of antihypertensive medications [10]. Treated hypertensive participants were considered controlled if they had an average SBP<140 mm Hg and DBP<90mm Hg. If the participants answered “yes” to the question: “Have you ever been told by your doctor that you had high BP?” and/or if they self-reported antihypertensive medication intake, they were considered as clinically diagnosed (aware) of their hypertension. All clinically undiagnosed or non-treated participants were thus considered as unaware of their hypertension.

#### Dyslipidemia

Subjects with lipid disorder (dyslipidemia) were defined as having at least one of the following anomalies: T-C≥190 mg/dl, TG≥150 mg/dl, LDL-C≥115 mg/dl, and HDL-C <40 mg/dl for men and <46 mg/dl for women [11], and/or taking lipid-lowering medications. A treated person was classified as controlled if his/her T-C<190 mg/dl, TG<150 mg/dl, LDL-C<115 mg/dl, and HDL-C≥40 mg/dl for men and ≥46 mg/dl for women. Similar to other pathologies, dyslipidemia participants were considered as clinically diagnosed (aware) of their lipid disorders if they reported that they had ever been told by their doctor that they had dyslipidemia and/or if they self-reported lipid-lowering medication intake. All clinically undiagnosed or non-treated participants were thus considered as unaware of their lipid disorders.

### Framingham Risk Score Calculation

For every participant, the Framingham risk score (FRS) to predict 10-year CHD risk was calculated using the adapted simplified model of Wilson et al. [12]. Those with personal history of myocardial infarction were excluded from the risk analysis. A risk of CHD greater than 20 percent in 10 years is considered high risk; intermediate risk ranges from 10 percent to 20 percent; less than 10 percent is considered low risk.

### Data Analysis

To account for the complex sampling design and for non-response, the prevalence rates were weighted to produce nationally representative estimates of the total population residing in Luxembourg, aged 18 to 69 years. The weights were calculated based on the lastly available national census data. All analyses were performed with PASW® for Windows® version 18.0 software (formerly SPSS Statistics Inc. Chicago, Illinois) and survey procedure for complex sampling design.

By using descriptive statistics, the level of management of hypertension, diabetes and dyslipidemia was compared to illustrate, via pie chart, the proportion of three groups: treated; non-treated subjects, in addition to those free of the conditions. Among treated cases, the proportions of controlled and uncontrolled cases were also presented.

Multivariable logistic regression analysis was performed to identify the independent contribution of health perception, socio-demographic and lifestyle factors to the risk of having hypertension or dyslipidemia but being unaware of these conditions. We measured several variables that might affect the likelihood of unawareness, including age, gender, country of birth (Luxembourgish; Portuguese; Europeans; non-Europeans), educational level (primary; secondary; tertiary), economical status (living above; below poverty threshold), family history of same pathologies (yes; no), BMI, and perceived health status (excellent/very good; good; and fair/poor), tobacco consumption (smokers; non-smokers), and regular family doctor (yes; no). The estimation of odds ratios and 95% confidence interval were computed for the unaware participants of their hypertension or lipid disorder.

We calculated the 10-year Framingham risk score for each participant and determined the proportion of people unaware of their increased risk of developing CHD or dying from heart attack within 10 years.

### Ethical Aspect

All participants were duly informed and provided their written consent prior to take part in the study, which was approved by the National Research Ethics Committee and the National Commission for Private Data Protection.

## Results

### Prevalence of Unawareness of Diabetes, Hypertension and Dyslipidemia among the General Population

The ORISCAV-LUX survey recruited 1 432 subjects, representing a total of 298 521 adults, aged 18 to 69 years, residing in Luxembourg. Table 1 displays that 4.4% (representing 12 667 subjects) have diabetes, 34.5% (representing 103 041 subjects) have hypertension and 69.9% (representing 205 367 subjects) have dyslipidemia. Among those diagnosed cases, respectively, 32% (representing 4 042 subjects) were unaware of their diabetes, 60% (representing 61 818 subjects) were unaware of their hypertension and 85% (representing 174 728 subjects) were unaware of their dyslipidemia.

**Table 1:** Prevalence of unawareness of diabetes, hypertension and dyslipidemia among the population in Luxembourg, ORISCAV-LUX 2007–2008.

	Diagnosed cases*		Unaware men		Unaware women		Total unaware subjects†	
Age, y	% (n)	Estimated population	% (n)	Estimated population	% (n)	Estimated population	% (n)	Estimated population
**Diabetes**	4.4 (69)	12 667	27.2 (10)	2 052	38.9 (12)	1 990	31.9 (22)	4 042
18–29 years	5 (1)	350	100.0 (1)	351	0.0 (0)	0	100.0 (1)	351
30–49 years	1.8 (14)	2 464	35.2 (3)	535	61.8 (4)	583	45.4 (7)	1 118
50–69 years	11.3 (54)	9 853	20.6 (6)	1 167	33.7 (8)	1 407	26.1 (14)	2 573
**Hypertension**	34.5 (540)	103 041	61.5 (196)	38 713	57.6 (122)	23 106	60.0 (318)	61 818
18–29 years	7.8 (17)	5 207	100.0 (10)	3 114	100.0 (7)	2 094	100.0 (17)	5 207
30–49 years	29.2 (218)	41 612	75.0 (108)	20 998	66.5 (46)	9 052	72.2 (154)	30 049
50–69 years	63.2 (305)	56 222	45.8 (78)	14 601	49.1 (69)	11 960	47.2 (147)	26 561
**Dyslipidemia**	69.9 (1033)	205 367	83.6 (439)	91 674	86.8 (432)	83 054	85.1 (871)	174 728
18–29 years	42.5 (92)	27 446	97.9 (43)	13 500	97.9 (47)	13 362	97.9 (90)	26 862
30–49 years	68.4 (495)	96 370	92.0 (255)	50 813	96.5 (208)	39 708	93.9 (463)	90 520
50–69 years	92.3 (446)	81 551	67.3 (141)	27 362	73.3 (177)	29 984	70.3 (318)	57 346

Data are presented as % (n).

* indicates the population having the following pathologies: diabetes, hypertension and dyslipidemia (including those aware and unaware).

† indicates the total population (men & women) unaware of their respective pathologies.

### Determinants of Unawareness of Hypertension and Dyslipidemia

Given the small number of participants unaware of their diabetes (only 22 cases), the results of logistic regression analyses are limited to the potential determinants of unawareness of hypertension and dyslipidemia. Increasing age and BMI were by far the strongest protective factors against the unawareness of both hypertension and dyslipidemia. There were no significant associations between gender, country of birth, economic status, education level, smoking habits and lack of awareness of hypertension and dyslipidemia. However, having a family history of similar conditions decreased independently the risk of unawareness of hypertension (OR = 0.57; 95% CI 0.36, 0.92; *P* = 0.021). In addition, not having a family doctor increased double-fold the odd of being unaware of hypertension (*P* = 0.048), whereas, poor health perception reduced significantly the risk of unawareness of lipid disorder (OR = 0.27; 95% CI 0.11, 0.68) (Table 2).

**Table 2:** Multivariable analysis of predictors of the lack of awareness of hypertension and dyslipidemia among the population in Luxembourg, ORISCAV-LUX 2007–2008.

			Lack of awareness of hypertension		Lack of awareness of dyslipidemia	
Indicators		Categories	OR (95% CI)	p-value	OR (95% CI)	*P*-value
Age, years	10 units		0.92 (0.88–0.95)	<0.0001	0.91 (0.89–0.93)	<0.0001
Gender		Men *v.* women	1.04 (0.66–1.64)	0.88	0.76 (0.49–1.17)	0.21
BMI		10 units	0.92 (0.88–0.95)	<0.0001	0.94 (0.90–0.98)	0.003
Country of birth		Europeans *v.* Luxembourgish	1.19 (0.64–2.23)	0.88	1.04 (0.60–1.80)	0.28
		Non-Europeans *v.* Luxembourgish	1.01 (0.32–3.14)		1.40 (0.43–4.62)	
		Portuguese *v.* Luxembourgish	0.84 (0.40–1.76)		0.50 (0.22–1.14)	
Economic status		Living below *v.* above poverty threshold	0.68 (0.40–1.18)	0.17	1.08 (0.59–2.06)	0.75
Education level		Primary *v.* tertiary	0.72 (0.36–1.44)	0.38	0.77 (0.39–1.54)	0.60
		Secondary *v.* tertiary	0.66 (0.37–1.19)		0.73 (0.40–1.34)	
Health perception		Average v. good/excellent	0.79 (0.49–1.27)	0.49	0.91 (0.57–1.44)	0.022
		Poor *v.* good/excellent	0.66 (0.27–1.62)		0.27 (0.11–0.68)	
Family history		Family history *v.* no family history	0.57 (0.36–0.92)	0.021	0.78 (0.50–1.22)	0.28
Family doctor		No *v.* yes	1.84 (1.004–3.38)	0.048	0.92 (0.51–1.66)	0.78
Smoking status		Smokers *v.* non-smokers	0.98 (0.50–1.90)	0.95	1.11 (0.65–1.90)	0.70

### Level of Management (Treatment and Control) of Diabetes, Hypertension and Dyslipidemia

**Figure 1:**
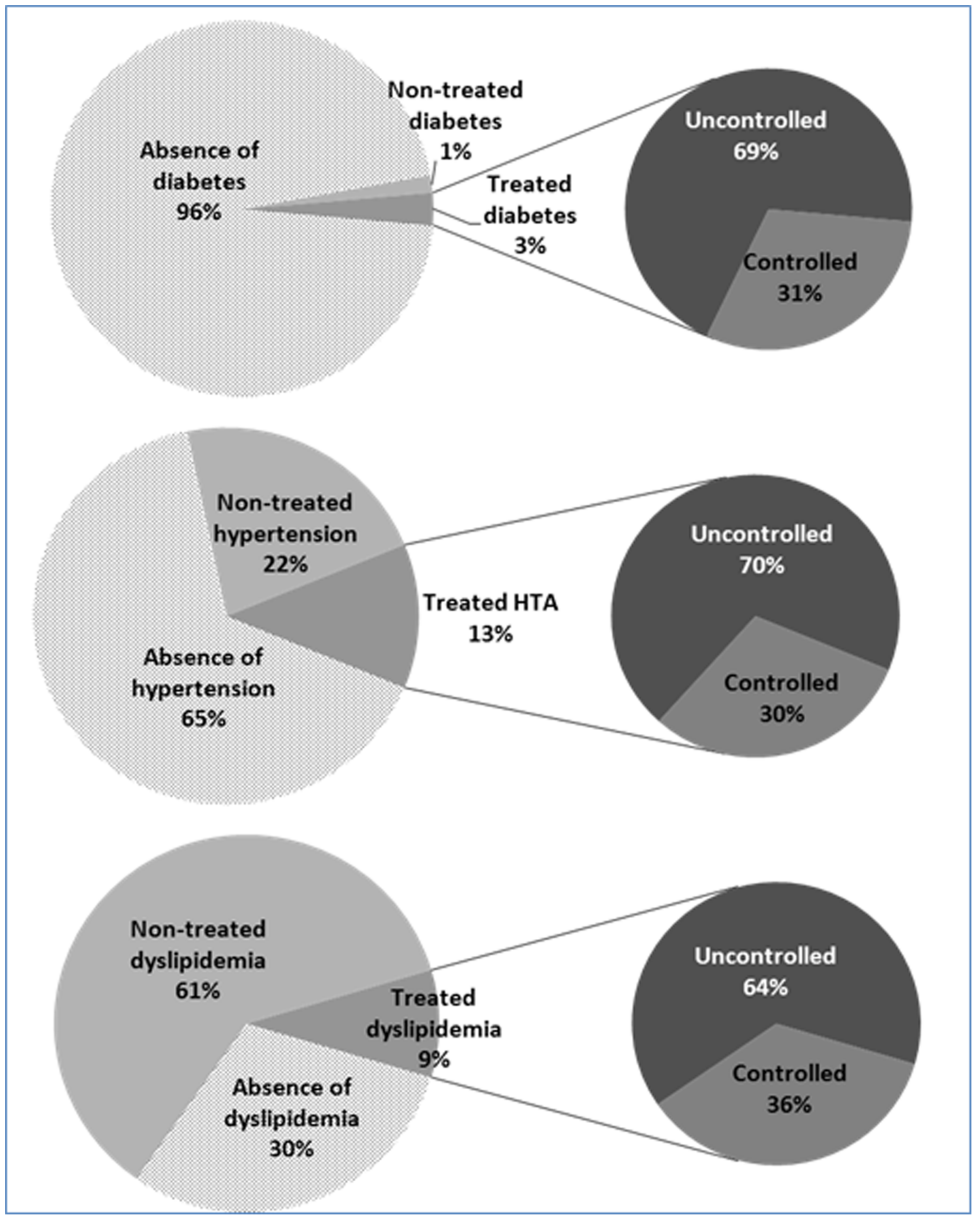
Proportion of non-treated, treated and controlled among treated subjects with diabetes, hypertension and dyslipidemia.

Figure 1 illustrates the proportions of participants free of diabetes, hypertension and dyslipidemia, the proportions of treated and non-treated participants, in addition to the controlled and uncontrolled cases (among treated participants) of their respective pathologies. Overall, about 4% of the studied population was diagnosed as diabetics, 35% as hypertensive and 70% as having dyslipidemia. Although diabetes was markedly less common, but it was better treated than hypertension and dyslipidemia. Among diabetic subjects, 3% were treated vs. 1% not treated. In contrast, 22% of the hypertensive participants were not treated vs. 13% treated. Concerning dyslipidemia, only 9% of those with lipid disorder were under drug medication vs. 61% not treated. For the treated cases of diabetes, hypertension and dyslipidemia, almost only one-third was under control.

### Exposure of Unaware Participants to 10-year Risk of Coronary Heart Disease

**Figure 2:**
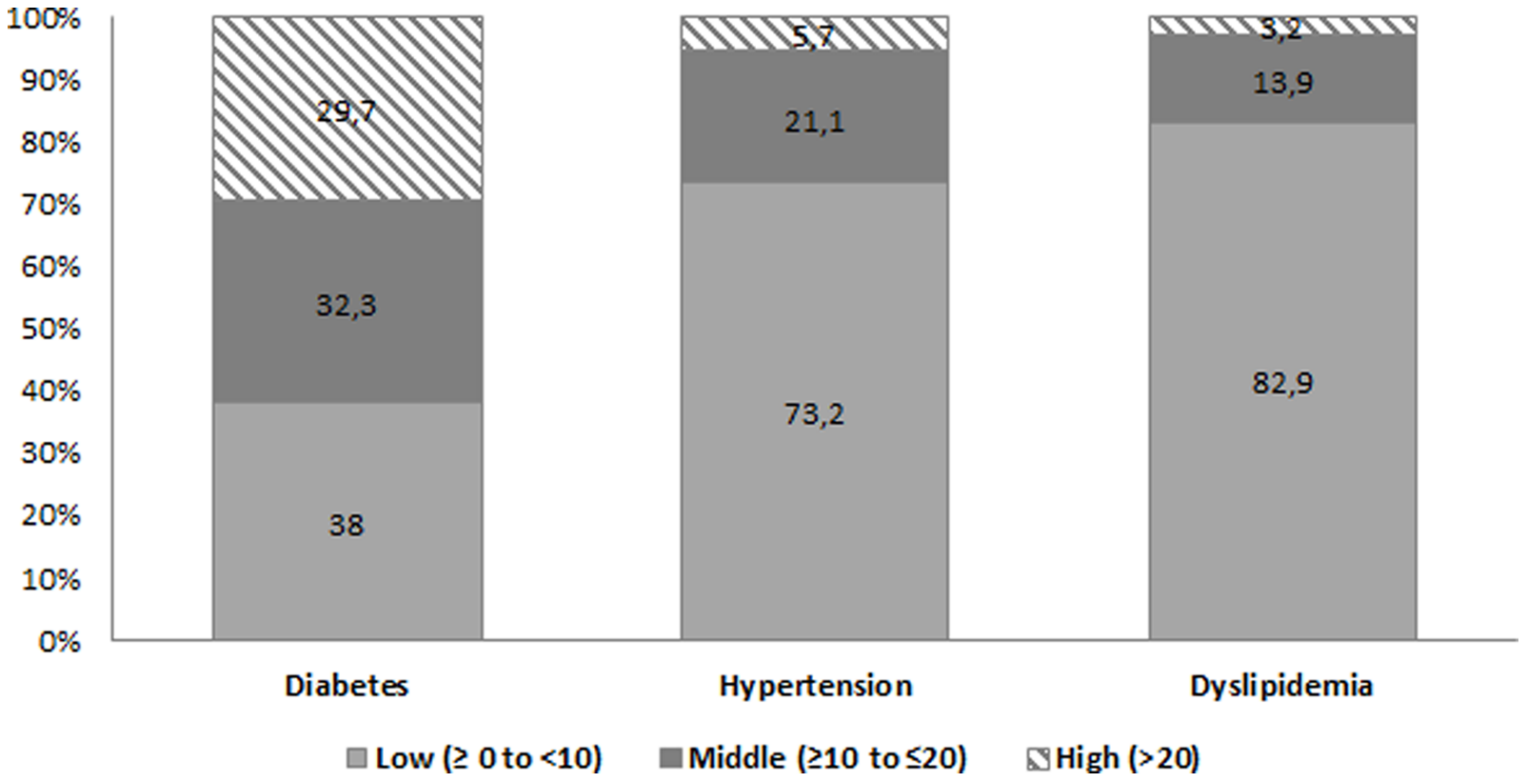
Distribution of unaware participants of their diabetes, hypertension and dyslipidemia, according to the level of 10-year Framingham risk score.

Descriptive results with regard to 10-year risk of CHD among the unaware participants of having diabetes, hypertension and dyslipidemia are shown in Figure 2. Among the unaware/untreated participants of their diabetes, 62% were at moderate to high risk, compared to 38% at low risk (less than 10 percent). About one-third of subjects, classified as hypertensive, but unaware and thus untreated and uncontrolled, had a 10-year Framingham risk score ≥10 percent. This group divided into two risk classes: 21% were at moderate risk and 6% percent were at high risk (>20 percent risk). Regarding the subjects unaware/untreated of their lipid disorder, although 83% were at low risk, about 17% were at moderate to high risk of developing CHD, within 10 years.

## Discussion

The main finding of the present national population-based study is the poor awareness of the general adult population to their affection by diabetes, hypertension and dyslipidemia. The estimated thousands of people, particularly of old age group (50–69 years) with these treatable and preventable cardiovascular risk factors, but unaware of their pathologies are worrying.

The incidence and prevalence of these chronic pathologies are expected to rise with the aging of the population. Undoubtedly, the development of prevention strategies and appropriate therapeutic control are important to decrease mortality, maintain quality of life and reduce the burden on healthcare resources. Although the awareness among our studied population increased concomitantly with age, the level of unawareness/non-treatment among the older age group was still exceedingly high (26% for diabetes, 47% for hypertension and 70% for dyslipidemia). Several factors could explain this high level of unawareness, such as the type of pathology (silent or late manifestation), debated diagnostic criteria (for example, thresholds of dyslipidemia), or unwillingness for health-monitoring. While dyslipidemia is almost asymptomatic and its detection requires blood analysis which must be prescribed by a physician, diabetes induces signs or symptoms leading the subject to seek medical care. Blood pressure measurement is usually part of clinical examinations, and can be detected in case of early consultation. Concerning the health seeking behaviors, the way in which Luxembourg healthcare system operates is similar to most European countries; it is based on the following fundamental principles: free choice of the provider by the patient and compulsory health insurance for the residents. This social model should help to reduce a potential delay to diagnose pathologies, improve treatment compliance and ultimately improve global health of the population. On the other hand, there are no particular health facilities for free-routine medical checkups. Therefore, health seeking-behaviors depend largely on the subject awareness for the importance to consult and monitor his health status.

More worrisome, approximately two-thirds of treated cases of diabetes, hypertension and dyslipidemia were not correctly controlled according to clinical guidelines. Numerous studies have revealed poor awareness and unsatisfactory treatment and control in many countries [13], [14], [15], [16], [17]. As in other European settings [18], our findings indicate that the management of these pathologies is far from being optimal. From a clinical and public health standpoint, intensive efforts to improve the awareness, treatment and control, particularly for hypertension and dyslipidemia should be considered among the priorities of national healthcare authorities and health professionals in the primary care sector. Increased patient awareness and compliance, together with increased adherence of primary-care physicians to current guidelines, may help to reduce the long-term cardiovascular events and mortality.

Regarding the determinants of unawareness, increasing age and BMI were the strongest protective factors against the lack of awareness of both hypertension and dyslipidemia. The plausible explanation is that elderly people become more worried by their health, particularly cardiovascular complications, than the young, who generally enjoy good apparent health and are less concerned by cardiovascular problems.

Likewise, obese subjects are more conscious of their cardiovascular health’s risk than the slim ones, probably because they are constantly in contact with primary health care providers to seek medical and dietary advice. These consultations increase their awareness of the underlying silent metabolic pathologies associated with excess body weight.

Consistent with these findings, the absence of family history was associated with lack of awareness of hypertension. Unsurprisingly, having a family member with such health problem increases the consciousness and alertness of the whole family for potential siblings’ affection.

On the other hand, poor health perception was associated with lack of awareness of lipid disorder; in the sense that subjects having a feeling of poor health were more aware of their pathology compared to those enjoying a good/excellent health perception. This finding is not surprising as subjective ill-health leads the individual to seek medical care and increases hence the likelihood to detect his/her pathologies.

In a similar study [17], having a usual health care provider (family doctor) was strongly associated with awareness and treatment of all these conditions, probably because these subjects consult more frequently than those without family doctor, then allowing early detection and treatment of their pathologies. In contrast to a similar American study [19], insurance coverage was not considered as relevant covariate, and hence not included in our multivariable logistic regression analyses as social security coverage in Luxembourg achieves nearly 96% of the total population with an absence of important healthcare access discrimination.

The American Cholesterol Education Program Expert Panel on Detection, Evaluation, and Treatment of High Blood Cholesterol in Adults recommends the determination of short-term (within 10 years) CHD risk as a means of assessing the need for intervention and/or prevention strategies [20]. The Framingham risk score is a well-recognized and validated tool for assessing the risk of experiencing a severe CHD event (angina pectoris, myocardial infarction or death) within 10 years, in individuals who have no clinically established cardiovascular disease [12]. In our study, an important proportion of unaware subjects were exposed to a likelihood of experiencing a CHD event within 10 years. These high-risk participants are asymptomatic and unaware of their risk, hence untreated and their pathologies were uncontrolled. Therefore, the development of effective strategies to prevent and properly manage these silent cardiovascular pathologies is of paramount importance.

This report represents the first reliable snapshot of the current situation as regards the level of awareness and management of three important preventable and treatable cardiovascular risk factors among the general population in Luxembourg. The analysis was based on a representative sample of adult residents, from whom extensive direct objective measurements were obtained. Additionally, the present study contributes to expand the body of knowledge regarding the epidemiology of these cardiovascular risk factors in Europe, particularly awareness, treatment and control rates.

Noteworthy, the comparison of known demographic and cardiovascular health-related profiles of the participants and non-participants to ORISCAV-LUX survey was demonstrated earlier [4]; the participants did not differ substantially from the non-participants, and the response rate allowed generalizing the findings for the entire population.

Similar to most nationwide epidemiological studies [18], [19], [21], [22], [23], the potential drawback of the present findings may be related to the single-occasion measurement of hypertension, which may yield some false-positive cases. However, the strict measures applied to obtain a true single-occasion hypertension should reduce the potential overestimation, for example: controlling for room temperature; avoiding white coat effect, refraining smoking prior to examination; allowing 30-min rest before at least 3 measurements by trained research nurse; and using the mean value of the last 2 readings for the analysis [6].

Besides, the remarkably high prevalence of dyslipidemia might be magnified due to the strict lipids thresholds, which were formerly suggested to identify subjects who would only need prophylactic medication and who otherwise would be considered at lower risk. Although the conflicting international treatment thresholds complicate comparison with previous studies [24], [25], [26], our percentages are among the highest reported in the literature [3]. This high prevalence of dyslipidemia calls for an urgent need to lipid disorder-focused prevention intervention and for an evaluation of current national nutritional recommendations.

In conclusion, this study highlights the considerable lack of awareness and insufficient management of the most important preventable and treatable cardiovascular risk factors (diabetes, hypertension and dyslipidemia). In a tiny country as Luxembourg (about 500 000 inhabitants), the overall estimated number of adults who have these pathologies but were unaware of their condition, untreated and uncontrolled exceeds two hundred thousand subjects. These findings provide a possible explanation of the steadily high cardiovascular mortality despite the clinical and therapeutic progress and accessibility. When calling to fight cardiovascular disease, by controlling treatable risk factors, the healthcare authorities and clinical community should be aware of the magnitude of efforts required to achieve this goal. The present findings indicate the urgent need for intensive efforts to reduce the gap in prevention strategies, via screening for asymptomatic pathologies since young ages, and control of clinical cases according to explicit clinical guidelines. Besides current hospital-based prevention and pharmaceutical control measures, mass education campaigns and lifestyle interventions are warranted. More emphasis should be given to the role of family doctor as a primary-care health provider.
